# Donor-Derived Human Parvovirus B19 Infection in Kidney Transplantation

**DOI:** 10.3389/fcimb.2021.753970

**Published:** 2021-10-15

**Authors:** Yedong Yu, Chunchun Wei, Junhao Lyu, Xiaoliang Wu, Rending Wang, Hongfeng Huang, Jianyong Wu, Jianghua Chen, Wenhan Peng

**Affiliations:** ^1^ Kidney Disease Center, the First Affiliated Hospital Zhejiang University School of Medicine, Hangzhou, China; ^2^ Kidney Disease Immunology Laboratory, State Administration of Traditional Chinese Medicine of China, Hangzhou, China; ^3^ Key Laboratory of Multiple Organ Transplantation, Ministry of Health of China, Hangzhou, China; ^4^ Institute of Nephrology, Zhejiang University, Hangzhou, China; ^5^ Key Laboratory of Kidney Disease Prevention and Control Technology, Zhejiang Province, Hangzhou, China; ^6^ Department of Intensive Care Medicine, the First Affiliated Hospital Zhejiang University School of Medicine, Hangzhou, China

**Keywords:** kidney transplantation, living donor, deceased donor, pure red cell aplasia (PRCA), human parvovirus B19

## Abstract

**Background:**

Donor-derived human parvovirus B19 (B19V) infections are rarely reported. Thus, its incidence in kidney transplantation is still unknown due to lack of surveillance studies. Similarly, whether the donor needs to be routinely screened for B19V and whether the kidneys from those with B19V DNAemia could be accepted also remain unknown.

**Methods:**

This retrospective study aims to evaluate the donor-derived B19V infections occurring in 823 living and 1,225 deceased donor kidney transplantations from January 2016 to December 2020. The serum viral load of living donors and their corresponding recipients was evaluated before and after transplantation. Meanwhile, for the deceased donor kidney transplantation, the serum viral load of recipients was only tested after transplantation; if recipients of a deceased donor subsequently developed B19V infection, the serum viral load of recipients and their corresponding donors before transplantation would then be further traced.

**Results:**

A total of 15 living donors were B19V DNAemia positive before the donation, of which B19V DNAemia occurred in three corresponding recipients. In deceased donor kidney transplantation, DNAemia occurred simultaneously in 18 recipients and their corresponding nine donors. A progressive decline in hemoglobin and reticulocyte count could be observed in one living donor recipient and other 11 deceased donor recipients, which were all well controlled by treatment eventually.

**Conclusion:**

The incidence of donor-derived B19V infection was 0.4% and 1.5% in living and deceased kidney transplantations, respectively. B19V was seemingly unnecessary to be routinely screened for the donor. Moreover, kidneys of the donors with B19V infection were acceptable.

## Introduction

Infection transmission from donor to recipient is one of the primary causes that affect early survival rate of recipients after transplantation (Kirchner and Pruett, 2016). The use of intensive immunosuppressive drugs in the early stage after transplantation may amplify the risk of infection carried by the donor organ (Fishman and Grossi, 2014). Many serious donor-derived infections caused by virus, such as rabies, West Nile virus, and lymphocytic choriomeningitis virus (Fischer, 2008), fall into unexpected infections category, with human parvovirus B19 (B19V) infections also being part of this category. Only a few cases have been reported regarding donor-transmitted B19V infections (Bertoni et al., 1995; Yango et al., 2002; Wasak-Szulkowska et al., 2008).

B19V is a small single-stranded DNA virus which is highly infectious and can cause a wide range of pathological conditions (Qiu et al., 2017). For immunocompromised patients, the most common clinical manifestation of B19V infection is pure red cell aplasia (PRCA) arising from the infection of erythroid progenitors in the bone marrow (Florea et al., 2007; Crabol et al., 2013). Furthermore, for kidney transplant recipients, B19V infection is associated with acute and chronic allograft injury (Barzon et al., 2009), antibody-mediated rejection, collapsing glomerulopathy (Moudgil et al., 2001), and thrombotic microangiopathy (Ardalan et al., 2008).

However, due to the lack of surveillance studies, the incidence of donor-derived B19V infections in kidney transplantations still remains unknown. In addition, a series of clinical issues regarding donor-derived B19V infections are all uncertain currently, such as whether the kidney donor needs to be routinely screened for B19V, whether the kidneys from the donor with B19V DNAemia are acceptable, what are the clinical features of donor-derived B19V infections, how to treat donor-derived B19V infections, etc. Thus, this study provides further information on addressing these problems mentioned above.

We carried out this study with the purposes of determining the incidence, clinical features, and treatment of donor-derived B19V infections in kidney transplant recipients (includes living and deceased donor kidney transplantations).

## Methods

This was a single-center, retrospective, cohort study with the aim of evaluating the donor-derived B19V infections occurring in living and deceased donor kidney transplantations. For living donor kidney transplantation, all donors and the corresponding recipients included in this study were related. Since January 2015, the use of executed prisoners as a source of organs for transplantation had been comprehensively terminated in China (Zhang et al., 2015). For deceased donor kidney transplantation, all donor kidneys come from voluntary public donations. This study was approved (Reference Number: 2018-777) by the Ethics Committee of the First Affiliated Hospital of College of Medicine of Zhejiang University according to the Declaration of Helsinki and Istanbul on Organ Trafficking and Transplant Tourism. Before recruitment, written informed consents were obtained from all donors and recipients.

### Patient Selection

From January 2016 to December 2020, we performed 927 living donor kidney transplants and 1,267 deceased donor kidney transplants in the Center of Kidney Disease, the First Affiliated Hospital, College of Medicine, Zhejiang University. The following exclusion criteria were applied for patients: 1) those who received an organ other than a kidney, 2) those who received ABO incompatible kidney transplantation, or 3) those whose blood specimens could not be retained. Demographics, clinical characteristics, laboratory findings, and outcomes of donors and recipients were collected from kidney transplantation electronic database and medical records.

### B19V Diagnosis

Commercial human parvovirus B19 real-time polymerase chain reaction (PCR) kit (Liferiver™, Shanghai ZJ Bio-Tech Co., Ltd.) was used for the detection and quantification of B19V-DNA in serum. The lowest detection limit for this test was 1 × 10^3^ copies/ml and the linear range of detection ranged from 2 × 10^3^ to 1 × 10^8^ copies/ml. A serum sample with viral load corresponding to ≥1 × 10^3^ copies/ml was considered to be positive.

### Blood Specimen Collection

The serum of the living donors and their corresponding recipients were evaluated for B19V-DNA on the day before transplantation and within 7 days after the transplantation, while the serum from recipients of deceased donor were evaluated within 7 days after the transplantation. Furthermore, the serum from recipients with unexplained anemia and reticulocytopenia within 6 months after kidney transplantation were also tested. In post-transplantation setting, if recipients of deceased donor developed B19V infection, blood samples that were collected within 24 h before transplantation from both recipients and corresponding donors would be further evaluated for B19V-DNA.

Serum creatinine and the estimated glomerular filtration rate (eGFR) of recipients with donor-derived B19V infections were checked prior to B19V infection, during B19V infection, and 12 months after B19V infection. The eGFR was estimated with the Chronic Kidney Disease Epidemiology Collaboration CKD-EPI formula.

### Immunosuppressant Regimen

Standard triple-drug maintenance immunosuppression includes tacrolimus (serum trough level between 5 and 10 ng/ml in the first half year after transplantation), mycophenolic, and prednisone. All patients were treated with methylprednisolone 10 mg/kg/day on days 0, 1, and 2. Dosage of prednisone was slowly tapered from 60 to 10 mg/day in 1 month after transplantation. All patients received induction with antithymocyte globulin (ATG) or interleukin-2 receptor antagonist (basiliximab). Basiliximab was given with a dose of 20 mg on the operation day and the 4th day after transplantation. From baseline, ATG was administered at a dose of 1 mg/kg/day and a maximum of five doses was given. All recipients received prophylactic ganciclovir treatment with a dose of 500 mg three times daily for 3 months post-transplant. A daily dose of 40 mg/200 mg to 80 mg/400 mg sulfamethoxazole/trimethoprim was applied for 9–12 months to prevent pneumocystis carinii pneumonia (PCP) except patients with sulfa allergies.

### Statistical Analysis

Hemoglobin, serum creatinine, and eGFR in this article were described in the form of mean values plus standard deviations (SDs). Viral load and time of B19V infections were described as median values with ranges. Categorical variables were presented as percentages. For normally distributed data, continuous comparisons between the two groups were conducted using Student’s *t*-test, while the non-parametric Mann–Whitney *U* test was used for data that were not normally distributed. Chi-square or Fisher’s exact test was used for categorical data. All statistical tests were two-tailed, and *p <*0.05 was considered statistically significant.

## Results

From January 2016 to December 2020, we performed 927 living and 1,267 deceased donor kidney transplantations. According to the exclusion criteria, 104 living and 42 deceased donor recipients were excluded in this study. Recipients were followed up for an average of 27.9 months.

### Human Parvovirus B19 Infections in Living Donors and Their Corresponding Recipients

Among 823 living donors, for 15 (1.8%), it was possible to detect B19V-DNA in serum samples before donation. The median viral load was 3.1 × 10^3^ copies/ml, ranging from 1 × 10^3^ to 1.5 × 10^4^ copies/ml. All 15 donors had no clinical manifestations of B19V infection such as fever, flu-like symptoms, weakness, dyspnea, arthralgias, rashes, and orthostatic hypotension during the follow-up period from 1 to 34 months. For these 15 corresponding recipients, B19V DNAemia (median, 2.8 × 10^3^ copies/ml) could be detected in three patients within 7 days after transplantation (**Table 1**). Hemoglobin and platelet counts of these three corresponding donors were both normal, and only two donors had mild leukopenia before donation. Without treatment, these three recipients had no clinical manifestations of B19V infection but with low-level DNAemia after transplantation. However, one recipient developed progressive anemia and reticulocytopenia and did not respond well to erythropoietin. Eventually, she was diagnosed with PRCA at the 24th day after transplantation (**Table 2**). Viral load of the serum samples increased from 2.8 × 10^3^ to 2.6 × 10^10^ copies/ml and the reticulocyte was 0.1%. In the process of infection, the lowest hemoglobin concentration was 7.4 g/dl. By adjusting for the immunosuppressant and administrating with immunoglobulin, her B19V DNAemia turned into negative at the 117th day after transplantation. In addition, the donor was her husband whose DNAemia was 2.2 × 10^3^ copies/ml before donation, and the detailed information is shown in **Tables 1**, **2**.

**Table 1:** Clinical characteristics of donor-derived B19V infection in living or deceased donors and their corresponding kidney transplantation recipients.

Donor no.	Age (years)	Sex	Relationship		Underlying disease	WBC (×10^9^/L)	Hgb (g/dl)	Plt (×10^12^/L)	Number of B19V copies before Tx (copies/ml)	Recipient no.	Age (years)	Sex	Cause of ESRD	Dialysis mode	Dialysis time (months)	Number of B19V copies before Tx (copies/ml)	Number of B19V copies within 7 days after Tx (copies/ml)
Living donor transplantation																	
A	18	M		Husband	None	8.4	15.4	210	2.2 × 10^3^	1*	44	F	GN	NO	/	Negative	2.8 × 10^3^
B	25	F		Mother	None	3.6	13.0	199	1.3 × 10^4^	2	36	F	IgAN	NO	/	Negative	1.8 × 10^3^
C	22	F		Mother	None	3.4	13.3	131	1.0 × 10^4^	3	25	F	GN	HD	4	Negative	4.6 × 10^3^
Deceased donor transplantation																	
D	18	M		/	MD	33.2	13.2	388	5.1 × 10^5^	4	49	M	GN	PD	37	Negative	1.2 × 10^4^
										5	31	F	GN	HD	44	Negative	1.1 × 10^4^
E	25	F		/	CR	15.7	7.1	262	1.0 × 10^10^	6*	34	M	GN	PD	54	Negative	7.3 × 10^9^
										7*	44	M	GN	PD	55	Negative	1.3 × 10^6^
F	22	M		/	CR	11.8	8.4	151	3.0 × 10^9^	8*	29	M	GN	PD	46	Negative	2.3 × 10^9^
										9	58	M	GN	HD	86	Negative	3.7 × 10^5^
G	37	M		/	CR	16.5	6.5	NA	1.4 × 10^4^	10*	30	F	IgAN	HD	50	Negative	4.8 × 10^6^
										11	51	M	GN	PD	10	Negative	3.6 × 10^3^
H	41	M		/	CR	4.0	5.4	102	1.2 × 10^4^	12	36	M	IgAN	PD	38	Negative	6.2 × 10^3^
										13	41	M	HBV-GN	PD	58	Negative	1.8 × 10^4^
I	61	M		/	CR	26.5	8.9	186	2.5 × 10^4^	14*	62	M	GN	HD	11	Negative	7.9 × 10^10^
										15*	40	M	GN	HD	89	Negative	8.5 × 10^10^
J	29	M		/	CR	8.8	5.7	347	2.3 × 10^10^	16*	55	M	GN	PD	55	Negative	7.5 × 10^7^
										17*	53	M	GN	PD	57	Negative	9.2 × 10^9^
K	65	M		/	CR	18.2	6.8	47	1.9 × 10^6^	18*	56	F	PK	HD	108	Negative	3.4 × 10^9^
										19	46	F	GN	PD	74	Negative	1.1 × 10^5^
L	32	M		/	CR	15.2	7.3	432	1.7 × 10^5^	20*	34	M	GN	HD	58	Negative	1.3 × 10^10^
										21*	43	F	FSGS	PD	57	Negative	1.9 × 10^5^

*The recipient developed pure red cell aplasia; the normal value of Hb ranges from 11.3 to 15.1 g/dl in female, 13.1 to 17.2 g/dl in male; WBC ranges from 4 to 10 × 10^9^/L; Plt ranges from 100 to 300 × 10^12^/L.

WBC, white blood cell; Hgb, hemoglobin; Plt, platelet; ESRD, end-stage renal disease; Tx, transplantation; GN, glomerulonephritis; IgAN, immunoglobulin A nephropathy; HBV-GN, HBV-associated glomerulonephritis; PK, polycystic kidney; FSGS, focal segmental glomerulosclerosis; NA, not available; HD, hemodialysis; PD, peritoneal dialysis; MD, muscular dystrophy; CR, craniocerebral trauma.

**Table 2:** Clinical characteristic of recipients with pure red cell aplasia (PRCA) caused by B19V after kidney transplantation.

	Recipients											
	Living donor	Deceased donor										
No.	1	6	7	8	10	14	15	16	17	18	20	21
Gender	F	M	M	M	F	M	M	M	M	F	M	F
Age, years	44	34	44	29	30	62	40	55	53	56	34	43
Number of B19V copies before Tx of the corresponding donor (copies/ml)	2.2 × 10^3^	1.0 × 10^10^	1.0 × 10^10^	3.0 × 10^9^	1.4 × 10^4^	2.5 × 10^4^	2.5 × 10^4^	2.3 × 10^10^	2.3 × 10^10^	1.9 × 10^6^	1.7 × 10^5^	1.7 × 10^5^
Number of B19V copies before Tx of recipients (copies/ml)	Negative	Negative	Negative	Negative	Negative	Negative	Negative	Negative	Negative	Negative	Negative	Negative
Number of B19V copies of recipients within 7 days after Tx (copies/ml)	2.8 × 10^3^	7.3 × 10^9^	1.3 × 10^6^	2.3 × 10^9^	4.8 × 10^6^	7.9 × 10^10^	8.5 × 10^10^	7.5 × 10^7^	9.2 × 10^9^	3.4 × 10^9^	1.3 × 10^10^	1.9 × 10^5^
Time to PRCA, number of days after Tx	24	7	7	7	7	7	7	7	7	7	7	39
Number of B19V copies of recipients at the day of PRCA diagnosis, (copies/ml)	2.6 × 10^10^	7.3 × 10^9^	1.3 × 10^6^	2.3 × 10^9^	4.8 × 10^6^	7.9 × 10^10^	8.5 × 10^10^	7.5 × 10^7^	9.2 × 10^9^	3.4 × 10^9^	1.3 × 10^10^	2.1 × 10^6^
Percentage of reticulocytes at PRCA diagnosis (%)	0.1	0.1	0.1	0.1	0.2	0.1	0.2	0.1	0.1	0.1	0.1	0.1
Hb at the lowest value, g/dl	7.4	5.9	8.4	8.7	6.1	4.4	6.6	8.6	5.5	5.8	7.2	4.6
Days after Tx	34	21	21	31	65	66	45	7	59	35	49	48
Treatment	IVIG	IVIG	Conservative therapy	IVIG	IVIG	IVIG	IVIG	IVIG	IVIG	IVIG	IVIG	IVIG
Number of B19V copies at the latest follow-up, copies/ml	Negative	Negative	Negative	Negative	Negative	2.0 × 10^3^	Negative	4.7 × 10^3^	1.5 × 10^3^	8.5 × 10^4^	3.6 × 10^4^	2.3 × 10^4^
Days after Tx	117	141	85	33	307	172	149	38	206	128	61	101
Months of follow-up	21.7	24.7	53.5	45.8	21.9	6.8	11.5	8.0	8.7	6.5	5.0	5.1
Hb at latest follow-up, g/dl	13.5	13.3	16.5	16.9	11.4	12.1	14.9	13.0	12.6	12.4	15.3	13.9

Among 823 recipients, 16 (1.9%) were found to be B19-DNA positive before transplantation although no B19-DNA could be detected in the serum from their corresponding donors. The median viral load was 4.45 × 10^3^ copies/ml, ranging from 2.2 × 10^3^ to 2.5 × 10^5^ copies/ml. The hemoglobin of 16 recipients raised from 10.3 ± 1.2 g/dl before transplantation to 11.4 ± 1.4 g/dl after 1-month transplantation. Two recipients with low-level DNAemia after transplantation had no clinical manifestation of B19V infection during follow-up. Moreover, B19V DNAemia of 14 recipients turned negative within 7 days after operation. However, one recipient was diagnosed with PRCA at the 41th day after transplantation. At that time, viral load of the serum sample was 6.06 × 10^9^ copies/ml and the reticulocyte was 0.1%. During the course of infection, the lowest hemoglobin concentration was 5.6 g/dl. Eventually, his B19V DNAemia turned into negative at the 510th day after transplantation by treatment.

B19V DNAemia, along with pure red cell aplasia, had occurred in 32 (3.9%) recipients at the median time of 42 days (ranging from 7 to 237 days) after transplantation, and their median viral load was 2.95 × 10^9^ copies/ml, ranging from 3.4 × 10^7^ to 1.1 × 10^11^ copies/ml. In addition, all of these recipients and their corresponding donors were both negative for B19V DNAemia before transplantations. In the process of infection, the mean lowest hemoglobin concentration was 6.4 ± 1.3 g/dl (ranging from 4.3 to 9.2 g/dl) and the reticulocyte was no more than 0.4%.

### Human Parvovirus B19 Infections in Deceased Donor Kidney Transplantation

Among 1,225 deceased donor kidney transplantations, 48 (3.9%) recipients who were B19V DNAemia negative before transplantation switched to positive within 7 days after the transplantation. The median viral load was 2.1 × 10^5^ copies/ml, ranging from 1.1 × 10^3^ to 8.5 × 10^10^ copies/ml. It was noteworthy that 9 corresponding donors in 18 of 48 recipients were B19V DNAemia positive, and the detailed information is shown in **Table 1**. Among the deceased donors, six had anemia and leukocytosis, two had anemia, and one had leukocytosis, but these results could be obscured by the primary disease. In addition, among 18 corresponding recipients, 11 were diagnosed with PRCA. By adjusting the dose of the immunosuppressant, the B19V DNAemia of one recipient (no. 7) turned into negative at the 85th day after transplantation. By adjusting the immunosuppressant and expanding the dose of immunoglobulin, the B19V DNAemia of four recipients turned into negative and six recipients with persistent low-level DNAemia had no clinical manifestation of B19V during follow-up, and the detailed information is shown in **Table 2**. The other seven recipients had no clinical manifestations of B19V infection. As described in **Table 3**, a total of 18 cases of donor-derived B19V infection were reported from deceased donor kidney transplantation, and there were no significant differences between PRCA recipients and non-PRCA recipients with respect to gender, age, dialysis mode, dialysis duration, and viral load of the corresponding donors before transplantation. However, the PRCA group had a higher B19V copy number within 7 days after transplantation than the non-PRCA group (*p* = 0.001).

**Table 3:** Comparison between the PRCA group with the non-PRCA group in a total of 18 donor-deprived parvovirus B19 infection in deceased donor kidney transplantation.

Total (*N* = 18)	PRCA (*N* = 11)	Non-PRCA (*N* = 7)	*p*-value
Gender, male %	8 (72.7)	5 (71.4)	0.676
Age, years, mean ± SD	43.64 ± 11.43	44.57 ± 9.25	0.438
Dialysis, PD%	6 (54.5)	5 (71.4)	0.637
Dialysis duration, days	58.18 ± 24.42	49.57 ± 25.44	0.544
Number of B19 copies before Tx in the corresponding donor (log10 copies/ml), P50 (P25–P75)	6.3 (5.0–10.0)	5.7 (4.1–6.1)	0.194
B19 within 7 days after Tx (log10 copies/ml), P50 (P25–P75)	9.5 (7.6–10.0)	3.8 (3.4–4.8)	0.001

Of the remaining 30 recipients who developed B19V DNAemia within 7 days after transplantation, 13 recipients were diagnosed with PRCA at the median time of 14 days (ranging from 7 to 48 days) after transplantation. The median viral load was 7.5 × 10^9^ copies/ml (ranging from 1.5 × 10^8^ to 2.3 × 10^10^ copies/ml) at the diagnosis of PRCA. Seventeen recipients had no clinical manifestation of B19V infection during follow-up. Additionally, B19V DNAemia of these corresponding donors was negative before transplantation. Interestingly, the allograft of eight recipients came from the same four donors.

Among the other 1,177 deceased donor kidney transplantations, B19V infection occurred in 14 (1.2%) recipients who were B19V DNAemia negative before and within 7 days after transplantation. Moreover, these corresponding donors were both B19V DNAemia negative before donation. For the other 14 recipients who received the same donor kidney, B19V DNAemia was also negative. The median time of infection was 30 days (ranging from 21 to 666 days) after transplantation, and the median viral load was 7.85 × 10^9^ copies/ml (ranging from 7.6 × 10^7^ to 6.8 × 10^10^ copies/ml). In the process of infection, the mean lowest hemoglobin concentration was 5.6 ± 0.6 g/dl (ranging from 4.9 to 6.8 g/dl) and the reticulocytes were no more than 0.3%.

As noted in **Table 4**, in the setting of donor-derived B19V infection after living or deceased donor kidney transplantations, the mean serum creatinine was 167.7 ± 77.0, 157.6 ± 68.2, and 110.8 ± 27.3 μmol/L prior to infection, during the infection, and 12 months after infection, respectively, and the eGFR was 48.5 ± 25.1, 51.3 ± 26.2, and 66.8 ± 18.9 ml/min/1.73 m^2^ prior to infection, during the infection, and 12 months after infection, respectively. In this study, recipients infected with B19V from the donor did not develop B19V-related graft damage that compromises allograft function. We can observe that transient elevated serum creatinine occurred in 6/21 (28.6%) recipients (nos. 1, 2, 16, 17, 19, 21). At the latest follow-up, these recipients were all alive with functioning allografts.

**Table 4:** The impact of B19V on allograft function in donor-derived infection after kidney transplantation.

Recipients, no.	Prior to B19V infection		At the time of B19V infection		12 months after B19V infection	
	Scr, μmol/L	eGFR, ml/min/1.73 m^2^	Scr, μmol/L	eGFR, ml/min/1.73 m^2^	Scr, μmol/L	eGFR, ml/min/1.73 m^2^
1	50	112.6	53	110.5	88	68.1
2	128	46.0	134	43.0	153	37.0
3	77	92.1	71	101.6	86	90.0
4	150	45.1	133	52.2	75	98.9
5	85	76.8	74	90.7	76	87.2
6	270	25.2	235	29.8	100	83.1
7	223	29.0	204	32.2	102	74.0
8	121	68.8	110	77.2	96	90.4
9	97	72.8	96	73.7	94	75.1
10	213	26.0	195	28.9	120	51.6
11	205	31.2	172	38.5	123	57.0
12	213	33.1	191	37.7	141	54.0
13	107	73.4	102	77.7	105	75.0
14	163	38.1	149	42.4	144	44.2
15	198	35.1	173	41.0	172	41.0
16	143	47.0	138	49.0	90	81.0
17	157	42.0	161	41.0	92	81.0
18	382	11.0	345	12.0	144	35.0
19	179	29.0	200	25.0	80	75.0
20	269	25.0	263	26.0	137	57.0
21	92	60.0	110	48.1	109	48.0
Mean ± SD	161.7 ± 77.0	48.5 ± 25.1	157.6 ± 68.2	51.3 ± 26.2	110.8 ± 27.3	66.8 ± 18.9

Scr, serum creatinine; eGFR, estimated glomerular filtration rate; SD, standard deviation.

## Discussion

Unlike bacterial infections, donor-derived virus infections are rarely reported in the past due to difficulty of diagnosis, other than the hepatitis B virus, the hepatitis C virus, and the cytomegalovirus (Trotter et al., 2017). This study firstly investigates donor-derived B19V infection through monitoring DNAemia of the donors and corresponding recipients before and after transplantation in either living or deceased donor kidney transplantations.

In our study, kidney recipients with B19V infection predominantly presented with unexplained anemia, reticulocytopenia, and no response to erythropoietin therapy, and bone marrow examination (characteristic morphological findings) is also an important diagnostic basis (Waldman and Kopp, 2007b). Recipients with B19V infection were treated with intravenous immunoglobulin (IVIG) 20 g/day for 5–7 consecutive days and adjusted for the immunosuppressant. In case of non-response to the first IVIG course or recurrence, another course of IVIG (20 g/day for 5–7 days) may be given. Recipients with low-level B19V DNAemia and no clinical manifestations of B19V infection were left untreated but underwent regular follow-up.

We found that 15 (1.8%) living donors and 16 (1.9%) living recipients with low-level B19V DNAemia had no clinical manifestations of B19V infection before transplantation. The incidence of B19V DNAemia in living donors and recipients was very low before transplantation, and even if it occurred, the level of DNAemia was too low to cause serious consequences. Therefore, it did not appear to be necessary for the living donors and recipients to do the routine B19V screening before transplantation. Of course, if anemia of donors and recipients could not be explained clearly, B19V screening is still needed.

For 32 living recipients with pure red cell aplasia, the B19V infection seemed to be irrelevant to their corresponding donors. B19V has been shown to be transmitted mainly through inhalation of infected aerosol droplets in immunocompetent individuals (Waldman and Kopp, 2007a; Eid et al., 2013). As there was no transfusion of blood products during the transplantation, viral reactivation and/or the respiratory tract could be the route of B19V infection for those recipients who received kidneys from B19V-DNA-negative donors (Qiu et al., 2017). Therefore, we concluded that it was necessary to screen for B19V among recipients with anemia and reticulocytopenia after transplantation.

Unlike screening in living transplantation, only recipients of deceased donor were routinely screened for the B19V within 7 days after transplantation. Eighteen (1.5%) recipients receiving the kidneys from the nine corresponding donors were positive for B19V DNAemia within 7 days after transplantation. By analyzing the pre-transplant blood samples of these recipients and their corresponding donors, we can conclude that the recipients were infected with B19V from the donor. Among these 18 recipients, 11 developed pure red cell aplasia. The viral loads of donors E, F, and J were more than 1.0 × 10^9^ copies/ml, and five of the six corresponding recipients developed pure red cell aplasia. Although not statistically significant, it was noteworthy that the viral load of the donor was seemingly related to the incidence of developing pure red cell aplasia in recipients. What is more, our study demonstrated that a higher B19V viral load in recipients within 7 days after transplantation was positively correlated with the incidence of developing pure red cell aplasia in recipients with donor-derived B19V infection in deceased donor kidney transplantation. Thus, we need to pay more close attention to these patients and perform treatment in time. Moreover, we recommend that timely treatment is necessary if the B19V viral load is greater than 1.0 × 10^6^ copies/ml in recipient within 7 days after transplantation, and conservative observation can be adopted if the B19V viral load is less than 1.0 × 10^6^ copies/ml. For blood transfusion, as the B19V can also be transmitted by blood or pooled blood products, immunocompromised individuals were at high risk of severe complications due to B19V infection from contaminated blood products. Therefore, in 2004, the U.S. Food and Drug Administration suggested that a serum sample containing more than 1 × 10^4^ copies/ml of B19V-DNA should be excluded because of the risk of transmission (Qiu et al., 2017).

Eid et al., in 2006, reported that allograft dysfunction after solid organ and hematopoietic stem cell transplant was observed in 10% of cases at the time of B19V disease (Eid et al., 2006). In kidney transplantation, graft loss/dysfunction occurred in 15.6% recipients but there was no death from B19V infection. Recently, there was a publication from Rattani et al. describing allogeneic bone marrow graft failure after B19V infection in two patients (Rattani et al., 2021). However, in our study, no allograft dysfunction was observed in 21 recipients with donor-derived B19V infection and only 6/21 (28.6%) recipients presented a transient elevation of serum creatinine. According to the literature reporting that B19V has an impact on allograft function, in 2019, Hai An et al. reported that graft dysfunction was observed in four out of nine (45%) kidney recipients with B19V-associated anemia (Hai An et al., 2019). What is more, in 2013, Xiao et al. suggested that recipients with B19V infection may develop delayed renal damage (Xiao et al., 2013). However, in our study, we did not observe any case of graft dysfunction in kidney recipients with donor-derived B19V infection. In our center, donor-derived B19V DNAemia was often detected within 7 days after transplantation in kidney recipients, while most of them showed a rapid decrease in serum creatinine levels in that period. What is more, recipients were monitored regularly for viral load, hemoglobin, reticulocyte, serum creatinine, etc., so that they can be treated timely. This may explain why graft dysfunction was rarely seen in our study.

All recipients with donor-derived B19V infections showed excellent prognosis, and the kidney graft survival rate at 1 year was 100%, which suggested that the kidneys from B19V-infected donors were acceptable for kidney transplantation. In particular, in cases where the donor had low-level B19 DNAemia, the recipients were less likely to have obvious clinical manifestations and the B19V could be quickly cleared even if the recipient was infected. If the donor had high-level B19V DNAemia, as well as the recipient, timely treatments including the adjustment of immunosuppressant and/or the infusion of large doses of immunoglobulin were needed to be given to the recipient (Egbuna et al., 2006; Jordan et al., 2011). This study also suggested that the donor-derived B19V infections could be detected within 7 days after transplantation in most occasions. As mentioned above, it was also unnecessary to perform routine screening for B19V among deceased donors before donation.

Several limitations in our study are worth declaring. First, the present study was limited by its retrospective design. Another limitation was that we did not routinely detect for B19V in deceased donors. B19V of the donor would be retrospectively tested only when B19V infection occurred in their corresponding recipients. What is more, serological assays for detection of IgM/IgG antibodies from the living donors were not performed before transplantation. This may explain why B19 DNAemia was found only in three recipients of living donor transplantation. Lastly, there is still a lack of robust evidence to confirm that the infection was 100% derived from the donor.

In this study, we firstly reported the epidemiological data of donor-derived B19V infections in kidney transplantation through routinely monitoring B19V infection in living and deceased donors and recipients before and after transplantation. Considering the low incidence of donor-derived B19V infection and that the infection can be well controlled by treatment even if it happened, it seems unnecessary to screen for B19V among the donors. We reached the conclusion that kidneys from donors with B19V infection were acceptable for transplantation. Moreover, the DNAemia of the corresponding recipient should be closely monitored and early treatment should be initiated according to the virus level. We recommended that timely treatment is necessary if the viral load is greater than 1 × 10^6^ copies/ml in recipients within 7 days after transplantation, and conservative observation can be adopted if the viral load is less than 1 × 10^6^ copies/ml. We hope that our work will provide some theoretical basis for future research and clinical practices.

## Data Availability Statement

The original contributions presented in the study are included in the article/supplementary material. Further inquiries can be directed to the corresponding author.

## Author Contributions

WP conceived the study. YY, CW, JL, XW, RW, HH, and JW performed the data collection and analyses. YY prepared the first manuscript draft. WP and JC provided major revisions and comments to the manuscript. All authors contributed to the article and approved the submitted version.

## Funding

This work was supported by the National Health and Family Planning Commission of the People’s Republic of China (WKJ-ZJ-1924), Basic Public Welfare Research Program of Zhejiang Province (GF20H150031), and National Natural Science Foundation of China (81770752, 81970651).

## Conflict of Interest

The authors declare that the research was conducted in the absence of any commercial or financial relationships that could be construed as a potential conflict of interest.

## Publisher’s Note

All claims expressed in this article are solely those of the authors and do not necessarily represent those of their affiliated organizations, or those of the publisher, the editors and the reviewers. Any product that may be evaluated in this article, or claim that may be made by its manufacturer, is not guaranteed or endorsed by the publisher.
